# Clinical, inflammatory, and immune differences between COVID-19 patients with and without cancer: A protocol for systematic review and meta-analysis

**DOI:** 10.1097/MD.0000000000023015

**Published:** 2020-11-06

**Authors:** Zhongyang Yu, Peipei Wang, Bailin Chen, Zihao Zhang, Jun Jiang, Yulong Zhuang

**Affiliations:** a Oncology Department, Dongfang Hospital, Beijing University of Chinese Medicine, Fangguyuan Rd; b School of Life Sciences, Beijing University of Chinese Medicine; c School of TCM, Beijing University of Chinese Medicine, Sunshine South Street, Fangshan District, Beijing; d Gynecology Department, Zhejiang University of Traditional Chinese Medicine First Affiliated Hospital, Youdian Rd, Shangcheng District, Hangzhou City, Zhejiang Province, China.

**Keywords:** cancer, coronavirus, COVID-19, meta-analysis, systematic review

## Abstract

**Introduction:**

The World Health Organization announce that novel coronavirus (COVID-19) is pandemic worldwide on March 11, 2020. In this pandemic, cancer patients are prone to become critically ill after being infected with COVID-19 due to special immune conditions, and cannot effectively benefit from the treatment plan designed for normal people. However, only a few literatures report the differences between cancer patients and normal people after being infected with COVID-19. There is no systematic review to evaluate the clinical, inflammatory, and immune differences between COVID-19 patients with and without cancer. The systematic review aims to summarize and analyze the clinical, inflammatory, and immune differences between them.

**Methods and analysis:**

We plan to conduct a systematic review according to the Preferred Reporting Items for Systematic Review and Meta-analysis Protocols (PRISMA-P) guidelines. Several databases (PubMed/MEDLINE, Embase, Web of Science, The Cochrane Library, CNKI, CBM, VIP, WanFang) were searched for relevant eligible observational studies on COVID-19 patients with cancer published from December 2019 to September 2020. Two researchers (Y.ZY and W.PP) will independently complete search strategy formulation, literature selecting, Information extraction, data collation, and quality assessment. The primary outcome will be the clinical characteristics differences between COVID-19 patients with and without cancer. Secondary outcomes will include immune function regulation characteristics such as T cell subset status, inflammation and other factors for COVID-19 patients with cancer. We intend to perform a meta-analysis of studies calculating odds ratio differences (Hedge g) for comparison in Forest plots and subgroup analysis after assessment of heterogeneity using *I*^2^ statistics based on compatibility on the basis of population and outcomes.

**Ethics and dissemination:**

We will use the information from published researches with no need for ethical assessment. Our findings will be published in a peer-reviewed journal according to the PRISMA guidelines.

**PROSPERO registration number:**

CRD42020204417

## 1 Introduction

In December 2019, China reported an infectious disease outbreak caused by severe acute respiratory syndrome coronavirus 2 (SARS CoV-2), which was subsequently named COVID-19 by the WHO.^[1,2]^ The epidemic first appeared in Wuhan, China, and then spread in more than 200 countries around the world.^[3]^ On March 11, the WHO announced a worldwide pandemic of COVID-19.^[4]^ The average incubation period of the COVID-19 pneumonia is 5.2 days.^[5]^ Meanwhile, the virus has been confirmed to be spread from person to person, mainly through close contacts by respiratory droplets produced when an infected person coughs or sneezes.^[6]^ The main symptoms after COVID-19 infection are fever, cough, fatigue, slight dyspnea, sore throat, headache, conjunctivitis, and gastrointestinal issues.^[7]^

Many studies have pointed out that COVID-19 infection can lead to immune disorders, induce abnormal cytokines and chemokines, and other inflammatory reactions to cause tissue damage.^[8]^ Clinical studies have shown that the total number of lymphocytes and T cell subsets reduced in the COVID-19 patient's system.^[9]^ Especially in patients with severe COVID-19 infection, the percentage of initial helper T cells increased, while the percentage of memory helper T cells and CD28+ cytotoxic suppressor T cells decreased.^[10]^ In addition, COVID-19 can also cause cytokine storm (CS), which leads to tissue damage,^[11]^ and severe patients will inevitably progress to acute respiratory distress syndrome (ARDS).^[12]^ Furthermore, the severity of COVID-19 has been confirmed to be related to the levels of pro-inflammatory cytokines and immune cell subsets.^[13]^

Because of the destruction of immune balance and inflammatory response caused by COVID-19, cancer patients are regarded as a vulnerable group in this epidemic, and the probability that serious symptoms happen to tumor patients is much higher than that to general COVID-19 patients.^[14]^ Moreover, compared with 2.3% in the general patients, the case fatality rate was 5.6% among cancer patients.^[15]^ Some studies indicated that cancer patients with COVID-19 infection who took immunotherapy and anti-inflammatory therapy showed a better prognosis.^[16–20]^ Therefore, it is very important to grasp the specific clinical, inflammatory, and immune differences between COVID-19 patients with and without cancer. On the basis of these differences, it is advisable to formulate suitable immune balance adjustment and active anti-inflammatory treatment programs to improve the prognosis of cancer patients injected by COVID-19.

Although several researches propose that there are differences in clinical manifestations, immunity, and inflammation between cancer and noncancer patients infected with COVID-19, some of the researches are criticized due to small sample size, limited clinical information, and high heterogeneity, potentially resulting in failure to analyze the specific differences objectively.^[21,22]^ Moreover, there is still a lack of strong immune and inflammatory data to support the choice of anti-inflammatory and immune drugs for cancer patients with COVID-19. Therefore, this systematic review intends to review the differences in clinical manifestations, various types of inflammatory cells, inflammatory factors, immune cells, immune factors, etc, between COVID-19 patients with and without cancer, so as to provide a solid theoretical basis for immunotherapy, anti-inflammatory treatment, anti-cancer treatment drug selection, and specific plan formulation for clinically COVID-19 tumor patients.

## 2 Method and analysis

### 2.1 Study registration

**Figure 1:**
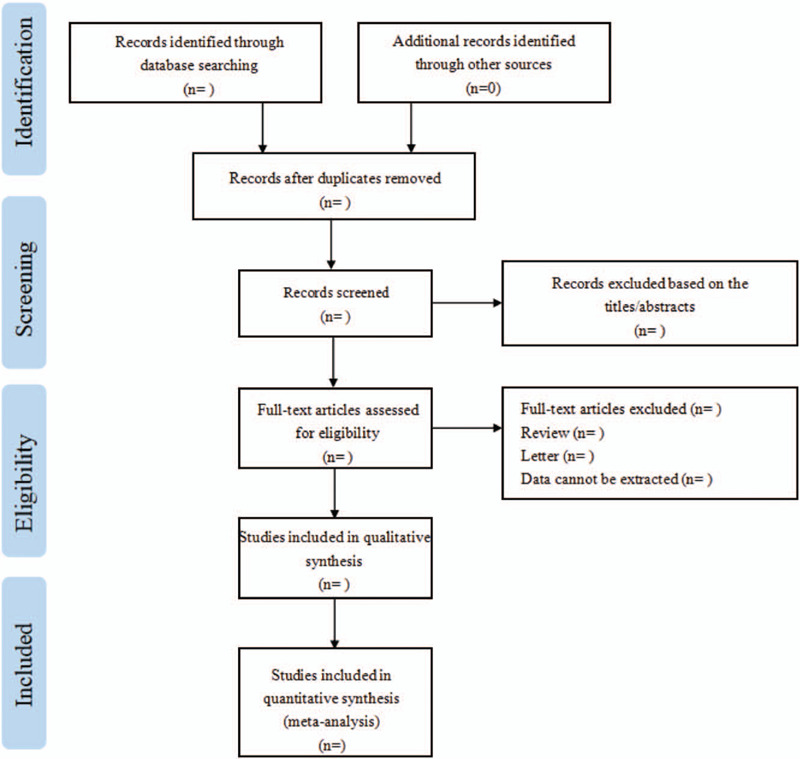
Flow diagram of the study selection process for the meta-analysis.

This systematic review was registered in the International Prospective Register of Systematic Reviews database (PROSPERO) on August 18, 2020 (CRD42020204417). This protocol was developed in accordance with the reporting guidance provided in the Preferred Reporting Items for Systematic Reviews and Meta-analyses Protocols (PRISMA-P) statement.^[23]^ In addition, Figure 1 represents the PRISMA flow diagram of our selection process. Meanwhile, we will update the PROSPERO record if there are any important amendments.

### 2.2 Eligibility criteria

#### 2.2.1 Inclusion criteria

*Design of studies:* This systematic evaluation research will include prospective/retrospective cohort studies, case--control studies, and cross-sectional studies to systematically characterize the clinical characteristics of cancer and COVID-19 patients and determine risk factors for disease severity.

*Participants:* The source of the cases was confirmed to be cancer patients with COVID-19 infection which is confirmed by RT-PCR laboratory, regardless of tumor types. The control group was nontumor patients with COVID-19 infection who lived in the same area with the case.

*Outcome:* The risk factors involved in the study included age, diet, tumor stage, family history of cancer, inflammatory response, and one or more changes of T cell subsets. Our primary outcome aims at clinical characteristics, inflammatory and immune status of COVID-19 infection in cancer patients.

*Range of studies:* Chinese and English literature published at home and abroad from December 01, 2019, to September 01, 2020. Meanwhile, the results of the studies can provide or be converted into odds ratio (OR), 95% credibility interval estimate (CI), and standard error (SE) data.

#### 2.2.2 Exclusion criteria

For the overall analysis of the clinical characteristics of cancer patients with COVID-19 infection, the following exclusion criteria apply: repeated publication; expert comments or case reports; abstracts of systematic reviews without published full-text papers, such as reference abstracts and letters to editors; reviews not published in English or Chinese; non-human or in vitro studies; incomplete information; and unable to complete data conversion.

### 2.3 Literature search and strategy

The main sources of literature search are database search, grey literature search, manual search, and citation search. An electronic search strategy was developed in collaboration with an experienced clinical research librarian using a method designed to optimize term selection. The databases we searched are PubMed/MEDLINE, Embase, Web of Science, The Cochrane Library, China national knowledge infrastructure (CNKI), China Biology Medicine disc (CBM), Vip Journal Resource Integration Service Platform (VIP), and Wanfang Data Knowledge Service Platform (WanFang). Two independent authors (YZY and WPP) conducted a systematic search on the eight databases above. The search strategy was based on “Tumor patients infected with COVID-19” and “clinical characteristics,” “risk factors” as keywords and entry-terms search, respectively. The details of PubMed search strategy are summarized in Table 1.

**Table 1:** Search strategy of PubMed.

Search	Query
#1	“COVID-19” [Mesh Terms]
#2	(((((((((2019 novel coronavirus disease[Title/Abstract]) OR (COVID19[Title/Abstract])) OR (COVID-19 pandemic[Title/Abstract])) OR (COVID-19 virus disease[Title/Abstract])) OR (2019 novel coronavirus infection[Title/Abstract])) OR (2019-nCoV infection[Title/Abstract])) OR (coronavirus disease 2019[Title/Abstract])) OR (coronavirus disease-19[Title/Abstract])) OR (2019-nCoV disease[Title/Abstract])) OR (COVID-19 virus infection[Title/Abstract])
#3	#1 OR #2
#4	“Neoplasms”[Mesh Terms]
#5	((((((((((((((((Neoplasia[Title/Abstract]) OR (Neoplasias[Title/Abstract])) OR (Neoplasm[Title/Abstract])) OR (Tumors[Title/Abstract])) OR (Tumor[Title/Abstract])) OR (Cancer[Title/Abstract])) OR (Cancers[Title/Abstract])) OR (Malignancy[Title/Abstract])) OR (Malignancies[Title/Abstract])) OR (Malignant Neoplasms[Title/Abstract])) OR (Malignant Neoplasm[Title/Abstract])) OR (Neoplasm, Malignant[Title/Abstract])) OR (Neoplasms, Malignant[Title/Abstract]))OR (Benign Neoplasms[Title/Abstract])) OR (Neoplasms, Benign[Title/Abstract])) OR (Benign Neoplasm[Title/Abstract])) OR (Neoplasm, Benign[Title/Abstract])
#6	#4 OR #5
#7	#3 AND #6
#8	(relative[Title/Abstract] AND risk^∗^[Title/Abstract]) OR (relative risk[Text Word]) OR risks [Text Word] OR cohort studies[MeSH:noexp] OR (cohort[Title/Abstract] AND stud^∗^[Title/Abstract])
#9	(“2019/12/01”[Date-Publication]: “2020/09/01”[Date-Publication])
#10	#7 AND #8 AND #9

### 2.4 Study screening and selection

First, use EndNote X9 software to classify and sort out the documents initially retrieved, and exclude the overlapping documents from different databases; Second, 2 independent authors (YZY, WPP) will read the bibliography, keywords, and abstracts of the documents, determine eligibility of studies for inclusion, and remove documents that obviously do not meet the theme; Third, according to the inclusion and exclusion criteria, review and screen the full text of the selected documents, and record the reason and number of the excluded documents; Fourth, carefully read the full text and tables of the further selected articles, and perform 3 screenings to analyze and determine documents such as repeated submissions, obvious errors in data, and excessively subjective evaluation criteria; fifth, extract data to the detailed data extraction tables. For incomplete information report, the original author can be contacted to supply the relevant materials, or it will be removed. In addition, if the observation indicators are not consistent or the data are missing and incorrect, researches will be excluded. If the clinical characteristics and risk factors are not described in detail or inconsistent, researches will also be excluded. The 2 independent authors (YZY, WPP) will review and screen several times, and the objections will be handed over to a third party for arbitration decision. Quality assessment will be performed on the relevant data of the included research literature, and the relevant research literature with similarity and high quality, which will be used for meta-analysis, is strictly screened. Finally, we will make a PRISMA document screening flowchart according to its screening process.^[24,25]^

### 2.5 Data collection processes

Data will be independently extracted by 2 review authors (YZY and WPP) using a pre-piloted data extraction form for collection of information in the following:

- Basic information: the first author's name, the year of publication.
- Description of study participants: Mean age, sex, geographic location, diagnosis and diagnostic system, number of participants, severity of tumor staging, study location and/or setting, and tumor complications, if any.
- Types of influencing factors and data: clinical characteristics, inflammatory and immune status clinicopathological features, and survival outcome of cancer and noncancer, and so on.
- Details of methodological: study design (prospective or retrospective), the population selection, comparability, and exposure.

Any discrepancies in the data extraction information will be reconciled by discussion among all the review authors and a joint check through the original papers will be adjudicated by authors YZY and WPP.

### 2.6 Assessment of methodological quality

In this study, 2 reviewers evaluated the quality of each study that met the inclusion and exclusion criteria. Quality assessments were performed based on Newcastle–Ottawa Scale (NOS), which is currently commonly used in the world to evaluate the quality of case–control studies and cohort studies including 8 questions and 3 items: population selection, exposure factor determination/outcome, and comparability between groups. Each item contains 4, 3, and 1 questions to evaluate the quality of the study. And the star rating is used. If it meets the requirements of the star rating, one “∗” is obtained, that is, 1 point is obtained, and the maximum score is 9 points. A study with score less than 4 is considered to be a low-quality and will not be accepted.

### 2.7 Data synthesis and analysis

All the tests of this study will use The Stata 16.0 software (Stata Corporation, TX). The χ^2^-based Cochrane Q test is used to analyze the impact of statistical heterogeneity, and *I*^2^ is used to evaluate the heterogeneity among the included studies. If *P* > .1 and *I*^2^ < 50%, which indicate that there is no statistical heterogeneity among the studies, we will try to use fixed-effect model. If *P* < .1 and *I*^2^ > 50%, which indicate a statistically significant difference, we will use a random-effects (DerSimonian--Laird method) model. At the same time, sensitivity analysis will be performed to analyze its heterogeneity. If there is significant heterogeneity in the results among studies, the reasons will be analyzed, or appropriate subgroup analysis will be performed according to the sources of possible heterogeneity. Considering the different types of tumors of study subjects, the included studies can be grouped according to the primary tumor to answer the clinical characteristics of patients infected with COVID-19 with different tumor types, and whether there is an interaction between the sub-combination effect size and the grouping factors. Funnel plot analysis will be used to determine whether there is publication bias. If data are missing, the data conversion will be carried out. A *P* value < .05 will be considered statistically significant.

## 3 Discussion

This systematic review and meta-analysis aims to analyze and summarize the clinical, inflammatory, and immune differences between COVID-19 patients with and without cancer. Our findings can provide a theoretical evidence for the guidelines of more effective and safer COVID-19 treatment plans for cancer patients who are considered as a vulnerable group during this global pandemic.

Our systematic review has a series of strengths. First of all, some of our project members personally participated in the fight against the COVID-19 epidemic, making our research closer to the clinic. Second, our review exhaustively and systematically analyzed existing literatures. Furthermore, we decide to contact with trial authors and ask for individual patient data in order to conduct a subgroup analysis.

However, this systematic review still remains the following limitations. First, our research only includes literatures written in English and Chinese, which may miss some potentially valuable journals in other languages. Second, we only reviewed researches published between December 2019 and September 2020. This may cause missed data.

This protocol based on the current research progress may have some shortcomings, but we will continue adjusting the research method during the implementation process and update our changes on our PROSPERO record in time.

## 4 Ethics and dissemination

There are no ethics approval required for this systematic review because we will use information from published researches. Our findings will be published in a peer-reviewed journal according to the PRISMA guidelines.

## Author contributions

Zhongyang Yu, Peipei Wang, and Bailin Chen designed the study. Zhongyang Yu, Zihao Zhang, Jun Jiang, and Yulong Zhuang drafted the manuscript. All authors approved the final manuscript.

**Conceptualization:** Zhongyang Yu.

**Data curation:** Zhongyang Yu.

**Formal analysis:** Peipei Wang.

**Funding acquisition:** Peipei Wang.

**Methodology:** Bailin Chen.

**Project administration:** Zhongyang Yu.

**Software:** Zihao Zhang.

**Supervision:** Yulong Zhuang.

**Validation:** Bailin Chen.

**Visualization:** Yulong Zhuang.

**Writing – original draft:** Zhongyang Yu.

**Writing – review & editing:** Zhongyang Yu, Jun Jiang.
